# High Serum Levels of TGF-β in Iranians With Chronic HBV Infection

**DOI:** 10.5812/hepatmon.7581

**Published:** 2012-11-05

**Authors:** Hossein Khorramdelazad, Gholamhossein Hassanshahi, Behzad Nasiri Ahmadabadi, Mohammad Kazemi Arababadi

**Affiliations:** 1 Molecular Medicine Research Center, Rafsanjan University of Medical Sciences, Rafsanjan, IR Iran; 2 Immunology of Infectious Diseases Research Center, Rafsanjan University of Medical Sciences, Rafsanjan, IR Iran

**Keywords:** Hepatitis B, Chronic, Transforming Growth Factor Beta

## Abstract

**Background:**

The transforming growth factor-β (TGF-β) is an important cytokine with anti-inflammatory properties.

**Objectives:**

The main purpose of this study was to compare the serum levels of TGF-β in a group of chronic HBV infected (CHB) patients as well as healthy individuals from South-East of Iran.

**Patients and Methods:**

Sixty patients with CHB as well as sixty healthy individuals were enrolled in the study. ELISA technique was applied to measure the serum levels of TGF-β in both groups.

**Results:**

Our results revealed that the serum levels of TGF-β were significantly increased in CHB patients in compare to healthy controls.

**Conclusions:**

According to this result, it may be concluded that high serum levels of TGF-β may be a mechanism by which immune response against HBV is suppressed.

## 1. Background

Iran is defined as a low endemicity part of the world for hepatitis B virus (HBV) infection, while, chronic form of the disease is frequent among HBV infected patients (1). Patients with chronic hepatitis B infection (CHB) are suffering from persistent HBV infection and the virus is not fully cleared from the hepatocytes (2). Several scientists proposed that the host genetic as well as epigenetic differences between patients and those who successfully clear the virus could be responsible for the prolongation of HBV infection (3, 4). Cytokines are small glycoproteins that are produced by immune cells and are involved in both inflammatory and anti-inflammatory reaction during diseases and heath (5). The TGF-β is a famous member of anti-inflammatory cytokine family that is produced during hemostasis and tissue remodeling (6). Therefore, it is plausible to hypothesize that its up or down-regulation may lead to inappropriate immune responses against viral hepatitis. Because, CHB patients are unable to completely eradicate HBV from their hepatocytes (1), a probable mechanism has been proposed in which chronic up-regulation of TGF-β cause’s decreased degree of immune responses to the infected hepatocytes (7). 

## 2. Objectives

Therefore, we aimed to determine the serum levels of TGF-β in the Iranian patients with CHB compared with healthy individuals.

## 3. Patients and Methods

### 3.1. Subjects

Peripheral blood samples were collected from 60 patients with CHB among with 60 healthy controls from Rafsanjan (South-Eastern of Iran) in 5.5 mL tubes free of anti-coagulant. Patients with detectable HIV antibody and HCV-HBV co-infected patients were excluded from the study. The patients were selected as CHB using the “Guide of Prevention and Treatment in Viral Hepatitis” criteria (8). Controls were selected with matched age and sex (Table 1). The samples were centrifuged at 3500 rpm for 4 min and serums were isolated immediately after collection. The serum samples were stored at -20 ºC for further cytokine analysis. This study was approved by the local ethical committee of the Rafsanjan University of Medical Sciences and informed written consent was obtained from all of participants, prior to sample collection.

**Table 1:** Laboratory and Demographic Information of CHB Patients and Healthy Controls

	CHB Patients	Healthy Controls
**Age, y, Mean ± SD**	35 ± 9	38.41 ± 7
**Liver function tests (LFT)**		
ALT, Mean ± SD	27 ± 12	28 ± 9
AST, Mean ± SD	28 ± 11	29 ± 5
ALP, Mean ± SD	270 ± 40	240 ± 20
**Sex**		
Male, No. (%)	28 (46.6%)	31 (48.3%)
Female, No. (%)	32 (53.4%)	29 (51.7%)

Abbreviations: ALT, Alanine aminotransferase; AST, Aspartate aminotransferase; ALP, Alkaline phosphatase; CHB, Chronic HBV Infection.

### 3.2. Detection of serological HBV markers and TGF-β

ELISA technique was used for samples HBsAg, HBeAg (Behring, Germany) and TGF-β (eBiosciences, Esp) screening using a commercial kit according to the manufacturer’s guidelines. Data were only used when the inter and intra-assays produced the scores of CV < 14% and CV < 3%, respectively. HBV-DNA Extraction and Real-time PCR condition: Viral DNA was purified from 200 μL of plasma from HBsAg positive patients using a commercial kit (Cinnaclon, Iran) according to the manufacturer’s guidelines. HBV-DNA quantification was also done using a commercial kit from Primer Design Company (UK) following the manufacturer’s instructions. Data analysis and statistical methods: The parametric statistical analyses were performed using the t-test under SPSS software version 18 and the P value of less than 0.05 considered as significant.

## 4. Discussion

**Figure 1:**
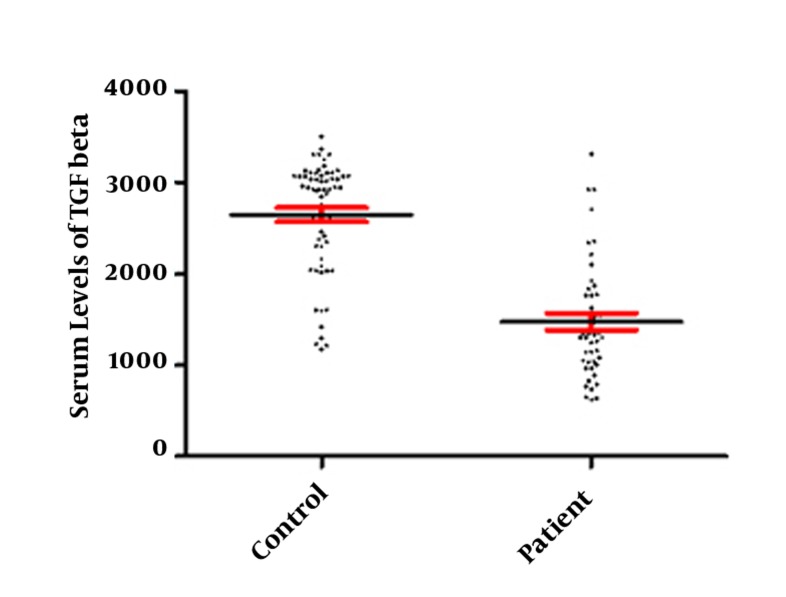
Demonstrates the Circulating Levels of TGF-β in the CHB Patients and Healthy Controls

**Figure 2:**
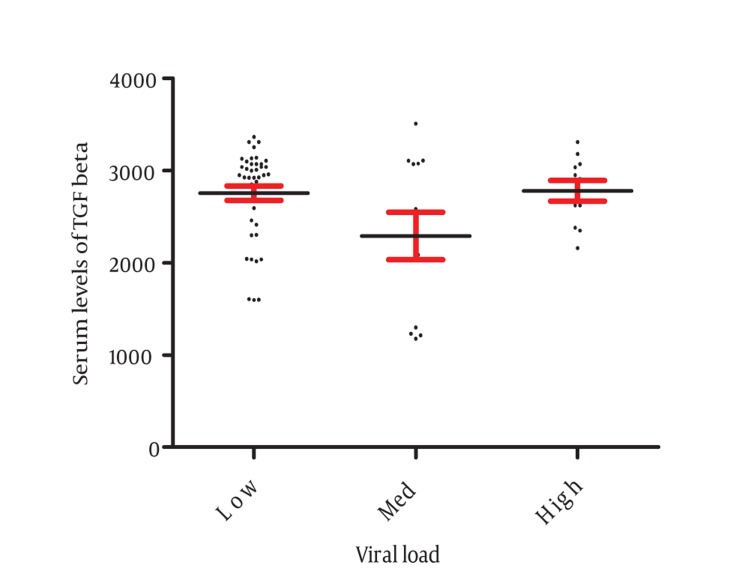
Demonstrates the Circulating Levels of TGF-β in the CHB Patients With Different HBV Viral Load

Our results revealed that all patients with CHB were positive for HBsAg with detectable HBV-DNA. Five (8.3%) CHB patients were HBeAg positive with high HBV-DNA copy numbers (more than 1000000 Copy/ml). The results also demonstrated that 37 patients had fewer than 20000, 12 between 20000-400000 and 11 upper than 1000000 copy numbers/mL. All patients and healthy controls had normal serum levels of AST, ALT, and ALP (Table 1). Results of this study showed that serum levels of TGF-β was significantly (P < 0.001) increased in CHB patients (2648.5 ± 77.35) in comparison with healthy controls (1476.56 ± 91.26) (Figure 1). The serum levels of TGF-β were not different from CHB patients with different HBV copy numbers (Figure 2). The cytokines network plays important role during immune response against intracellular infections including viral infection (9). TGF-β, as an anti-inflammatory cytokine, plays key roles in the regulation and suppression of immune responses (6). Therefore, any significant alteration in the expression of TGF-β may lead to inappropriate immune responses against viral infections. Our results demonstrated that the expression of TGF-β was increased in the CHB patients in comparing to healthy controls. Although, immune responses is complex and evaluation of a cytokine cannot be considered as whole feature of this system but based on our results it may be concluded that elevated expressions of TGF-β may be in parallel with immune responses suppression, hence, the disease can be maintained in the CHB patients. Additionally, TGF-β can induce apoptosis in hepatocytes (10); hence, the mechanisms which induce cirrhosis may be related to high levels of this cytokine in CHB patients. Interestingly, a previous study reported that TGF-β down-regulated the NKG2D/DAP10 and 2B4/SAP expression in human NK cells (7), thus this could be plausible proposed mechanism via it. TGF-β may suppress immune responses in CHB patients. In agreement with present findings, Divella et al. indicated that serum levels of TGF-β were higher in patients suffering from liver diseases (10). Another study that was undertaken on the Egyptian population also indicated that serum levels of TGF-β were increased in the hepatocellular carcinoma patients (11). Interestingly, Gue et al. reported that the level of TGF-β was significantly increased in the serum and liver tissues of the HBV infected patients with the progressive of fibrosis (12). Enhanced serum levels of TGF-β in CHB patients also were reported by Lebensztejn et al., (13) and Akpolat et al., (14). Moreover, the polymorphisms within the TGF-β gene were also showed to be associated with CHB (15). Additionally, the immune response process during HBV infection is complicated (16) and is highly related with the stage of hepatitis, hence, evaluation of several aspects of immune responses of CHB patients in different clinical presentation of HBV infection can be helpful to find out the main mechanisms involving the suppression of immune system during HBV infections. On the other hand, previous studies demonstrated that the immune response process is related to hepatocyte function (17, 18). Our results revealed that the serum levels of AST, ALT and ALP were not different between CHB patients and controls, hence, it seems that the hepatocyte function was unable to alter immune responses of the CHB patients and higher serum levels of TGF-β is related to the other mechanisms. On the other hand, our results showed that the serum levels of TGF-β were not different from CHB patients with different HBV copy numbers. Therefore, by the other means, our results may at least confirm that the production of TGF-β was not affected by virus replication. In contrast to our results, Li et al. revealed that HBcAg induces TGF-β production in CHB patients (19). Therefore, studies with larger sample size of CHB patients with CHB including inactive carriers as well as patients with more advance degree of liver disease are required to unravel the underlying mechanisms of probable association of TGF-β expression with HBV replication.
